#  Working Memory Training in the Form of Structured Games in Children with Attention Deficit Hyperactivity Disorder

**Published:** 2016-10

**Authors:** Fatemeh Khalili Kermani, Mohammad Reza Mohammadi, Fariba Yadegari, Fatemeh Haresabadi, Seyed Mehdi Sadeghi

**Affiliations:** 1 Department of Psychology, Islamic Azad University, Rudehen Branch, Tehran, Iran.; 2 Psychiatry and Psychology Research Centre, Roozbeh Hospital, Tehran University of Medical Sciences, Tehran, Iran.; 3 Department of Speech Therapy, University of Social Welfare and Rehabilitation Sciences, Tehran, Iran.; 4 Department of Speech Therapy, Mashhad University of Medical Sciences, Mashhad, Iran.; 5 Department of Industrial Design, Islamic Azad University, Central Tehran Branch**, **Tehran, Iran.

**Keywords:** *Attention Deficit*, *Hyperactivity*, *Structured Games*, *Working Memory Training*

## Abstract

**Objective:** In this study, a new training method of working memory (WM) was used in the form of structured games, and the effect of training was evaluated with a controlled design. The training method of WM in the form of structured games includes 20 sets of structured games that can improve WM and performance of executive functions.

**Method:** Sixty children with attention deficit hyperactivity disorder (ADHD) aged 8.5 to 11.2 years (35 boys), using no stimulant medication were selected. We randomly assigned 30 participants to the experimental group and provided them with WM training. The training was in the form of structured games and was offered to the participants in two 60-minute sessions weekly for 12 weeks. Other participants were assigned to the control group, receiving no treatment. All the participants were also evaluated at follow-up 6 months later. The main measures were the Child Behavior Checklist (CBCL), the Digit Span and Symbol Search B subscale of the Wechsler Intelligence Scale for Children (WISC-IV); and scores of dictation and mathematics were used in terms of pre and post-test.

**Results:** The results of the t-test revealed a significant improvement in the post-test measures as well as a significant reduction of parents’ reports of inattentiveness, and improvement in academic performance in the experimental group. However, no significant changes were found in the control group.

**Conclusion**: The academic and working memory improvements were primarily due to the training method of WM. Our findings suggest that the training method of WM in the form of structured games may be a practical method for treating children with ADHD, but it needs to be further investigated.

Attention deficit hyperactivity disorder (ADHD) have been observed in 3 to 5% of school-age children and resulted in impaired academic performance and social function and usually persist into adulthood (1, 2). This research on ADHD children is mostly based on two theoretical approaches: Working memory (WM) (3-9), and motivational variables (10, 11). 

According to the applied studies, WM is introduced as a core deficit in ADHD (12). WM is a limited capacity system for briefly storing and manipulating information. WM underlies the capacity to perform complex tasks such as learning, comprehension and planning (13, 14). WM is usually regarded as a fundamental function for other executive functions. Deficiencies in WM are mostly associated with some disorders and problems such as learning disabilities (15, 16), hyperactivity (9), lower academic performance (17-19), mathematical difficulties and classroom inattention (20). 

Recently, a computerized-training program has been developed to improve working memory deficits in children with ADHD, which consists of a number of computerized exercises (21-23). Motivation is a key component in memory training so the coach helps the person to maintain motivation throughout the training by continuous feedback and support (24). Computerized-training has only achieved moderate success in faster reaction times (25), retaining a large number of digits (26), improving WM in children with ADHD and ameliorating ADHD symptoms (23, 27) and in teaching rehearsal strategies to children with learning disabilities (2) and children with fetal alcohol spectrum disorder (28). However, no increase has been reported in WM capacity, long-lasting effects and generalization from trained tasks to non-trained tasks (23, 27).

A second approach to ADHD assumes an unusual sensitivity to reinforcement and reward (29-31). In fact, given the correct degree of external incentive during a potentially boring task may increase activation and effort resulting in cortical stimulation and helps children with ADHD to optimize their motivational state which lead to improve their performance (32) and withholds impulsive and inappropriate behaviors (33). Based on the cognitive-energetic model, ADHD causes defects at three levels: Cognitive mechanisms, energetic mechanisms and management system deficits. The cognitive-energetic model contains three levels. The first level of the cognitive-energetic model contains four general stages of encoding, search, decision and motor organization, which are cognitive mechanisms of attention. The second level comprises effort, arousal and activation, which are energetic mechanisms (34). The third level of the model is a management system associated with planning, monitoring and error detection and correction. These levels are depressed in ADHD (35). Based on cognitive-energetic model, children with ADHD suffer from non-optimal energetic behaviors (33). Reinforcement and motivation by means of games (36) or providing a game format for each task may stimulate the essential energy for completing the tasks (37, 38), and result in improvement in person’s performance of the cognitive tasks (33). Providing external motivating contingencies in children with ADHD increase the activation/arousal state and consequently promote their optimal cognitive performance (39, 40). These motivational variables were used in this study as basic features for ADHD children. In fact, the therapist- child relationship was one of the important variables, which was considered in this study. It might be assumed that this relationship plays a role in reinforcing and motivating children during the therapeutic process. 

In this study, we examined the effect of a novel WM training in the form of structured games in ADHD children in which the participants practiced structured games for twelve weeks. The key features of this training were using various techniques involving WM and executive functions on the one hand and the therapist -subjects’ relationship to encourage them to complete the games on the other. WM training in the form of structured games is appealing to children and enhances their motivation and attention. Due to the existence of a strong link between ADHD and academic impairment (41, 42), the dictation and mathematic scores of the participants were recorded (not part of the daily training) to find whether the training effect generalized to non-trained tasks before and after practice. Children in the experimental group practiced 20 structured games. Conducting the games was facilitated by the therapist’s encouragement. This procedure was hypothesized to optimize the performance of WM and executive functions. No intervention was conducted for the control group during the study period. In fact, the control group received therapeutic intervention after the study. By having two groups, we intended to estimate the effect of improvement of WM. To evaluate the effect of training, we used several measures that were not part of the training program.

## Materials and Method

***Participants***

An experimental study in the form of a randomized controlled trial (RCT) was carried out at three psychiatric centers in Tehran in 2013. Of 82 patients aged 8.5 to 11.2 years who referred to the psychiatrists in psychiatric centers, 60 were selected for this study and were randomly assigned into two experimental (n = 30) and control groups (n =30), using the Randomizer form to instantly generate random numbers (Table 1). Diagnostic assessment was confirmed by the physician, taken from the parents, based on the DSM-IV criteria (43). Informed consent was obtained from all the participants.

The inclusion criteria were: (a) Meeting DSM-IV diagnostic criteria for ADHD; (b) Age between 8 and 11 years at the start of the intervention. Exclusion criteria were (a) Being treated with stimulants such as atomoxetine, neuroleptic, or any other psychoactive drugs; (b) Fulfilling the Diagnostic criteria for oppositional defiant disorder, autistic syndrome, Asperger’s syndrome, conduct disorder, bipolar disorder, obsessive–compulsive disorder, tic or depression based on the psychiatrist diagnosis; (c) IQ <80 (based on the WISC-IV and school history); (d) Medical illness requiring immediate treatment. 

***Material***

A structured clinical interview was conducted with the parents based on the DSM–IV diagnostic criteria. To assess changes during the treatment, some tests were conducted in the form of pre and post-test. 

The Measure of Attention Deficit: The Child Behavior Checklist (CBCL) was used to assess the changes in the field of attention deficit (AD) and ADHD symptoms during the treatment. This checklist was a questionnaire completed by parents and indicated a large amount of children's behavioral problems in the form of T-scores. Accomplished studies suggested a correlation between CBCL's items and DSM-IV disorders (44). Minaei (2006) reported that internal consistency and test-retest reliability for CBCL were good, with Cronbach Alphas ranging from 0.77 to 0.83 (45, 46). 

The Measure of Verbal WM, Verbal WM was assessed using the Wechsler Intelligence Scale for Children (WISC-IV) Digit Span subtest (47, 48).

The Measure of Visuospatial WM, Visuospatial WM was assessed using the WISC–IV Symbol Search B subtest (47, 49). 

***The Scores of Dictation and Mathematics***

It was the scores of dictation and mathematics prior to the beginning of the intervention.

***WM Training in the Form of Structured Games***

The WM training in the form of structured games was designed and applied by a psychologist, one of the authors, according to her clinical experience in working with ADHD children. This method suits children 5–15 years of age. The program comprises of 20 different games with various techniques, involving WM and executive functions. 

WM operations consist of some kinds of transformation or manipulation of information (50), which was applied in this study.

a) Converting data into coded form of long-term memory in the form of basic and complex encoding. Basic encoding transforms perceptual input into suitable codes for short- or long-term storage and complex encoding associates meaningful information with related schemas stored in long-term storage (50). During this process, long-term knowledge is applied to recall and improve WM representations. Along with this, WM promotes the building and retrieving of long-term structures (51). This technique is applied using Sequence of Geometric Shapes, Continued Placement and Increasing Objects games, which will be introduced later. 

b) Linking new data to the existing long-term representations: During acquiring schemas and other long-term representational structures, new information is encoded and added to already existing schemas. Grouping discrete items into larger units or chunks and associating them with stored familiar patterns in long-term memory and enriching the organizational structure of long-term memory are used to extend WM span (50). This technique is applied in “Continued Placement game.

c) Maintaining the sub-products of calculative procedures until the last product is obtained: This process is facilitated by rehearsal which is a periodic repetitive process of holding information in WM for a longer course of time (52). This technique is applied in Continued Visuospatial Memory, Fruits Classification, Sequence of Geometric Shapes, Continued Placement and Increasing Objects games, which are introduced later.

d) The technique of managing a conscious, direct investigation for information collected in the long-term memory: This technique is applied in Continued Visuospatial Memory, Fruits Classification, Sequence of Geometric Shapes, Continued Placement and Increasing Objects games. (

f) Chunking related items into categories or groups: Chunking enriches the organizational structure of long-term memory by grouping same items, and then relating them to a familiar scheme in memory (53). This technique is applied in Fruits Classification game, which is introduced later. 

g) Organizing new information in form of meaningful categorization (54, 55): This process is done by mnemonics, which associate partly meaningless input with stored meaningful images or words in long-term memory. Mnemonics supply structure, meaning, integration and cues where none naturally exist, and consequently encoding and retrieval are facilitated (56). Some types of mnemonics included in this study were Ode/Rhyme, Image, visual imagery and First-letter Mnemonics (57). They are applied by several methods such as recoding or transformation of the information, additions to or elaborations of the material and a systematic retrieval component (58). This technique is applied using the games of Fruits Classification, Sequence of Geometric Shapes, Continued Placement and Increasing Objects.

These exercises varied in their inherent complexity and the program adjusted the level of difficulty for each exercise to the child’s ability to constantly challenge the capacity of the child’s working memory. 

***Procedure***

To improve WM performance, structured games were conducted in a period of 12 weeks consisting of two 60-minute sessions weekly. Selecting games in each session was done based on the conducted assessments. Based on each child’s readiness in playing the games, the games were repeated in other sessions. No intervention was done for the control group. In fact, the control group received treatment at the end of the study, which was not assessed nor entered into this research. 

The psychologist, one of the authors, practiced the designed games for the parents. After conducting each game, parents were trained to become familiar with the method of playing the games at home, supervising and encouraging their children to make active their thinking process. Parents were encouraged to conduct games at an easy level at home and gradually make them harder to motivate the child to continue the games. To assess the process of the therapy, we asked the parents to report to us the process of intervention at home. 

Simple instruments such as animals and the cards with the pictures of fruits, memory cards, color boxes and diagrams were used for playing. The basic level of each child was determined by using short-term visual memory (based on age, various cards were shown to the child. After naming them, the cards were taken and the child was asked to name them, auditory memory (WISC-IV Digit Span subtest) and visual attention (modeling third pattern of cubes).

As the definition and description of all 20 sets of structured games is a very long protocol, the entire process will be introduced in another study in the form of a therapeutic protocol. The protocol will provide a table for each of the tasks, defining the task demands, number of items, time to completion, responses required and constructs tapped. Some of these games are explained briefly as follows: 

1. Continued placement: The therapist put 10-40 cards on the table. The number of cards depended on the children’s evaluated skills and age. Then the therapist named some cards, and asked the child to put them in a specific place. The child had to select the cards and put them in their place. After the child put the cards in the place, the therapist asked the child to name those cards without looking at them; this shaped the mental imagination of the room in the child’s mind. The therapist named another group of cards and followed the game, using the same rules as the previous step. 

To facilitate the memorizing procedure, the therapist helped the participant to activate the performance of WM, using some strategies such as verbal rehearsal, chunking, adding details, encoding, organization and imagination. The number of cards and stages of the games had to be determined based on the basic level of the participant’s ability. At the end, the child gained the ability of naming all cards and their locations. 

2. Continued Visuospatial Memory: In this game, the therapist put two cards in two rows on the table and asked the child to name them, and then turned the cards and asked the child their names. After that, she added two more cards and after naming and turning them, the child named them. The therapist gradually increased the number of cards to 12, and followed the same rule for each series of cards. In the first step, the child named cards in a vertical form, but in the second step, he or she named them in a horizontal form (for changing the method of the brain processing). In the third step, the therapist added another 12 cards, which were similar to the first 12 cards and put them randomly in two rows above the two first rows, and asked the child to find the same cards in two first rows by taking one of the cards from the two-second rows. In the fourth step, the child needed to take the same two cards together. Organization and visual imagery strategies were used in this part of the game. In the fifth step, the child named all the cards in a sequence. The sixth step was conducted like the fifth step without any cards. 

3. Sequence of Geometric Shapes: In this game, the child was asked to arrange a row of geometric shapes in a sequence after memorizing each card (each card contained five geometric colorful shapes). In the second step, the child were reminded of all the previous cards which he/she memorized gradually. To facilitate this step, the therapist guided the child to encode each card with various methods such as first-letter mnemonics. 

4. Fruits Classification: In this game, the fruits were categorized based on their colors. Then they were covered with relatively similar geometric shapes, which increased visual attention and imagery. The child memorized what was put under the each geometric shape. As the covered fruits were gradually increasing, the therapist and the child reviewed them. At the end, the child could be able to name all of the covered fruits.

All the games used in this study affected WM and executive functions. The continuous processing of information was done under the supervision of the chief executive. Episodic buffer binds separate modules of information into chunks and integrates elements into new relevant structures. The visuospatial sketchpad, a passive temporary store and an active rehearsal process were involved in these games. Using some strategies such as sub-vocal verbal rehearsal, chunking (controlled by the episodic buffer), adding details, meaning-based encoding, organization, mnemonics and visual imagery, WM performance was improved in these games.

## Results

After the experimental group received the interventions, the first set of analysis was conducted on both groups to assess the effectiveness of the intervention in the experimental group compared with the control group. After six months, the scores of dictation and mathematic were analyzed in both groups to reassess the maintenance of changes. 

***The Measure of Attention Deficit***

Independent and paired sample tests revealed statistically significant changes in the experimental group in two items from the CBCL: Attention deficit (AD) and ADHD symptoms, (p = 0.0 for between and within the group comparison in attention deficit and ADHD symptoms). However, there were no significant differences in both symptoms score changes in the control group (P > 0.05). 

***The Measure of Verbal WM***

The differences in post-test scores of WISC-IV Digit Span subtest (47) were meaningful in the experimental group (Independent sample t-test: p = 0.0; paired sample test: p = 0.001) in contrast to the control group (P > 0.05). 

***The Measure of Visuospatial WM***

In the post-test of the WISC–IV Symbol Search B subtest (47), significant changes were observed in the experimental group (p = 0.0 for between and within the group comparison), which were not meaningful for the control group (P > 0.05).

**Table1:** The Characteristics of Experimental and Control Groups of ADHD Children

**Participants**	**Experimental**	**Control**
Sex (n (%))		
Male	18 (60)	17(56.66)
Female	12 (40)	13 (43.33)
Age (mean) (Range)	9.9 (8.5-11)	9.8 (8.11-11.2)
school grade		
% grade 3	43.33%	43.33%
% grade 4	33.33%	40%
% grade 5	23.33%	16.66%

**Table2:** Means, SDs, t and P-Value between and within Two Groups for the Psychological Assessment and Rating Scales

**Measures**	**Group**	**Mean and SD**		**“t” and p value** **(Within Group Comparison)**	**“t” and p-value ** **(between Group Comparison)**			
		**1**	**2**		**3**		**4**	
		**M ± SD**	**M ± SD**	**t (p)**	**t (p)**	**F (Sig.)**	**t (p)**	**F (Sig.)**
VWM	E	6.4000 ± 2.17051	13.8000 ± 4.98422	-4.935 (0.001)	0.929 (0.365)	0.551 (0.467)	4.604 (0.0)	5.326 (0.033)
	C	5.6000 ± 1.64655	6.2000 ± 1.54919	-1.964 (0.081)				
VS WM	E	7.6000 ± 1.2649	12.000 ± 1.3333	-12.944 (0.000)	0.758 (0.458)	1.558 (0.228)	7.426 (0.0)	0.009 (0.927)
	C	7.0 ± 2.16025	7.2000 ± 1.54919	-0.557 (0.591)				
ADHD	E	73.2000 ± 5.09466	45.0000 ± 6.84755	15.456 (0.000)	0.308 (0.762)	0.030 (0.865)	-10.173 (0.000)	1.023 (0.325)
	C	72.5000 ± 5.08265	72.6000 ± 5.16828	-130 (0.899)				
AD	E	81.5000± 4.24918	43.6000 ± 7.51591	14.136 (0.0)	0.052 (0.959)	0.012 (0.915)	-13.543 (0.0)	2.886 (0.107)
	C	81.4000 ± 4.29987	81.8000 ± 4.80278	-0.459 (0.657)				

df for within group comparison = 29, df for between group comparison = 58, 1= pre-assessment, 2= post-assessment, 3= pre to pre-assessment, 4= post to post-assessment, V WM = verbal working memory, VS WM = Visuospatial working memory, ADHD = attention deficit and hyperactivity, AD = attention deficit, E = experimental group, C = control group

**Table3:** Means, SDs, t and P-Value between and within the Two Groups for the Scores of Dictation and Mathematics

**Measures**	**Group**	**Mean and SD**			**“t” and p value (within Group)**			**“t” and p value (between Group)**					
		**1**	**2**	**3**	**4**	**5**	**6**	**7**		**8**		**9**	
		**M ± SD**	**M ± SD**	**M ± SD**	**t (p)**	**t (p)**	**t (p)**	**t (p)**	**F (Sig.)**	**t (p)**	**F** **(Sig.)**	**t (p)**	**F** **(Sig.)**
Dic.	E	17.3000± 2.00278	19.3000± 0.94868	19.4000± 0.69921	-5.071 (0.001)	-4.358 (0.002)	-0.429 (0.678)	0.689 (0.499)	0.118 (0.735)	4.350 (0.0)	3.985 (0.061)	6.199 (0.0)	10.083 (0.005)
	C	16.7000± 1.88856	16.2000± 2.04396	15.1000± 2.07900	1.627 (0.138)	4.311 (0.002)	6.128 (0.000)						
Math.	E	15.2000± 2.14994	18.4000 ± 1.17379	18.7000 ± 1.25167	-7.686 (0.0)	-8.174 (0.0)	-1.406 (0.193)	0.344 (0.735)	0.596 (0.450)	5.047 (0.0)	4.891 (0.040)	5.905 (0.0)	5.044 (0.038)
	C	14.9000± 1.72884	14.0 ± 2.49944	13.4000 ± 2.54733	2.212 (0.054)	3.503 (0.007)	1.964 (0.081)						

df for within group comparison = 29, df for between group comparison = 58, 1 = pre assessment, 2 = post assessment, 3 = post 6-month assessment, 4 = pre to post assessment, 5= pre to post 6 months assessment, 6= post to post 6 months assessment, 7=pre to pre-assessment, 8= post to post-assessment, 9= post 6 months to post 6 months assessment, Dic = scores of dictation, Math =scores of mathematic, E = experimental group, C = control group

***The Scores of Dictation and Mathematics***

The scores of the dictation and mathematic for experimental and control participants were assessed prior to the therapy and after applying games to see whether the training effect generalized to non-trained tasks. At the end of the intervention, meaningful changes in the scores of the experimental group were observed (Independent sample t-test: p = 0.0; paired sample test: p = 0.001 in dictation; p = 0.0 for between and within the group comparison in mathematic). 

In contrast, these changes were not found in the control group (P > 0.05) (Tables 2 and 3).

***Six-month Follow-up***

To gather information on the stability of the effects of the intervention, the scores of the dictation and mathematics of both groups were recorded after six months. Although the assessment was done in the second half of the academic year, it involved an increase in the content of the lessons. Furthermore, the score of dictation and mathematic in the experimental group was evaluated as high or intermediate. Additionally, parents reported suitable changes in the field of attention. In contrast, no difference was found in scores of children in the control group and parents’ reports in the field of attention (Table 3).

## Discussion

The results of this RCT study suggested that the novel WM training in the form of structured games may be a viable method to help ADHD children and has a good effect on performance of WM in the experimental group, and improves their academic performance, whereas these changes were not clear in the control group. 

The striking differences between WM training in this study and other researches are in the type of games and method of implementation. In this study, we investigated the effect of a novel WM training in the form of structured games in ADHD children. In contrast, the computerized-training program consists of a number of computerized exercises (21-23). 

Because of using various techniques, which are involved in the performance of WM and executive functions, WM training in the form of structured games directly enhances the effect of WM training. Outcome changes in WM of ADHD children in the experimental group were attributed to the following reasons: 

In designed games, the participants were gradually asked to do some tasks, memorize each step, review and express them. This continuous processing of information was done under the control of the chief executive and finally led to improved classroom attention and completing continues acts in participants (59). In some games, the participants asked to memorize the location of things, which were gradually increased. Reviewing this information engaged visuospatial sketchpad that is responsible for the short-term storage of visual and spatial information, generation and manipulation of mental images (60). Rehearsal process, oral presentation of information, gradual revising and reviewing information involved the phonological loop, which stored information in a phonological form.

Using strategies such as sub-vocal verbal rehearsal, chunking, adding details, meaning-based encoding, organization, mnemonics and visual imagery led to reinforcement of WM performance during plays (61), for example, chunking the enriched organizational structure of long-term memory by grouping same items, and then relating them with familiar scheme in memory (53). Rehearsal enhances short-term recall and facilitates encoding long-term storage, organization and categorization information by facilitating later recall new information and processing information in a more deep level. Mnemonics provide cognitive cuing structures in the form of visual images, sentences or rhymes (58). In addition, the existing bilateral relation between WM and long-term memory increase the expectation that reinforcing long-term memory resulted in high performance of WM. This process was applied by a gradual review of information and transferring them from short-term to long- term memory. 

Before and after practice, the participants’ scores of dictation and mathematic were recorded, which were not part of the WM training. Based on the finding, it was concluded that WM training in the form of structured games affects generalization to non-trained tasks. According to educational and psychological researches, there is a severe relation between measurements of WM and mathematics performance (62), such as mathematics problem solving (63). Executive WM appears to be responsible for coordinating, monitoring and sequencing all of the processing steps involved in mathematical procedures (64, 65). Estimating, counting and executing problem-solving strategies (66) play an important role in all types of mathematic tasks (64, 65). Based on these findings, it can be concluded that improving WM, especially executive WM, has had a positive impact on the performance of the experimental group in mathematics scores. 

In this study, using structured games affects children’s motivation and performance. In fact, game setting tasks improve motivation and optimal cognitive performance in ADHD children and prevent unsuitable and impulsive behaviors in them. Therefore, ADHD children have abnormal sensitivity to reinforcement (29, 31); reinforcement contingencies including rewards and accurate feedback have a positive impact on the task performance and motivation of children (33). This finding is in line with Prins study in which the game condition, compared to a control condition without game elements, yielded more impact on WM as measured by CBTT (11). In Prins study, the effects of game sets on motivation and performance of ADHD children were assessed in the form of standard computerized WM task (11), which was different with our study. 

The important point, which was not mentioned in the some previous studies, especially in computerized settings, is the role of patient-therapist relationship based on psychological variables. In this study, the therapist tried to establish a relationship with the participants, show respect and accept them, which are introduced as important aspects of treatment. In contrast, children also tried to respect the therapist and listen to her. Additionally, therapist’s feedbacks made the participants so motivated to complete the games and follow directions. Consequently, children’s self-efficiency was developed and they changed their belief to try more to reach more achievement. Raising self-efficacy led the participants to complete the tasks and continue therapy sessions, while they became tired because of difficulty of exercises. The main role of the therapist was teaching necessary techniques to the participants to complete the tasks, helping them to complete the tasks and giving them the feedback that their achievement resulted from their endeavor. Although the therapist played an important role in conducting the games and motivating children, her role was limited to increasing children’s motivation, which had no effect on improving the performance of WM and executive functions. This finding will be discussed in another study in which the author studies the effect of the therapist on children’s performance. 

The presence of parents in the therapy room and monitoring steps of therapy helped them to establish a more effective relationship with their children. The therapist provided the parents with the necessary recommendations, which made the role of the parents as significant as a therapy co-worker. Establishing a suitable parent-child relationship resulted in paying appropriate attention to the child, which is one of the essential needs of children. 

Memory interventions need to occur at critical developmental stages and before maturing of the specific regions of the brain. To succeed in education and brain reconstruction, purifying and treatment should be provided at the critical revolutionary levels (67). Once neural structures are established and myelination is completed, changes become more difficult. To increase the opportunity of success in memory interventions, it was suggested that interventions be applied in early childhood and early elementary years. However, higher-level cognitive processes that involve executive functions continue to develop throughout adolescence. In addition, as WM underlies so many types of learning in school, it is better to apply early intervention. Considering these facts, to reach better conclusion according to the brain flexibility, the participants were selected from elementary years.

## Limitation

Beyond descriptive outcomes, the conclusions are obviously limited by the relatively small sample size, limited assessment scale and limited range of ages. It would be important to replicate this research in larger, and in an extended range of age to ensure the efficacy and generalizability of such approach. Using other sub-tests of the WISC-IV can highlight the effect of intervention on other abilities. It would also be important to examine whether applying this method in the form of structured games affects inattention or hyperactivity– impulsivity and whether the therapist relationship with the children functions as modifiers of treatment effects, meaning that if other therapists conduct these games, it could be expected to reach the same results. In addition, it is recommended that this method be concurrently applied and compared with another method of reinforcing WM.

## Conclusion

The results are promising with regards to the use of WM training in the form of structured games on performance of WM, academic performance, inattention and hyperactivity symptoms in children with ADHD. The strength of this study was its method application. Designed structured games are appealing for children and enhance their motivation and attention. In addition, they are easy to apply, and parents or even teachers can easily perform this method in every situation. Overall, our study may have a wide range of implications for future developments of new, innovative and feasible interventions for children with ADHD or even normal children for increasing performance of WM as well as other mind skills. 

## References

[1] Biederman J, Mick E, Faraone SV (2000). Age-dependent decline of symptoms of attention deficit hyperactivity disorder: impact of remission definition and symptom type. Am J Psychiatry.

[2] Rasmussen P, Gillberg C (2000). Natural outcome of ADHD with developmental coordination disorder at age 22 years: a controlled, longitudinal, community-based study. J Am Acad Child Adolesc Psychiatry.

[3] Dowson JH, McLean A, Bazanis E, Toone B, Young S, Robbins TW (2004). Impaired spatial working memory in adults with attention-deficit/hyperactivity disorder: comparisons with performance in adults with borderline personality disorder and in control subjects. Acta Psychiatr Scand.

[4] Karatekin C, Asarnow RF (1998). Working memory in childhood-onset schizophrenia and attention-deficit/hyperactivity disorder. Psychiatry Res.

[5] Kempton S, Vance A, Maruff P, Luk E, Costin J, Pantelis C (1999). Executive function and attention deficit hyperactivity disorder: stimulant medication and better executive function performance in children. Psychol Med.

[6] Kuntsi J, Oosterlaan J, Stevenson J (2001). Psychological mechanisms in hyperactivity: I Response inhibition deficit, working memory impairment, delay aversion, or something else?. J Child Psychol Psychiatry.

[7] Mariani MA, Barkley RA (1997). Neuropsychological and academic functioning in preschool boys with attention deficit hyperactivity disorder. Dev Neuropsychol.

[8] Westerberg H, Hirvikoski T, Forssberg H, Klingberg T (2004). Visuo-spatial working memory span: a sensitive measure of cognitive deficits in children with ADHD. Child Neuropsychology.

[9] Karatekin C (2004). A test of the integrity of the components of Baddeley's model of working memory in attention-deficit/hyperactivity disorder (ADHD). J Child Psychol Psychiatry.

[10] Sagvolden T, Sergeant JA (1998). Attention-deficit hyperactivity disorder-from brain dysfunctions to behaviour. Behav Brain Res.

[11] Prins PJ, Dovis S, Ponsioen A, ten Brink E, van der Oord S (2011). Does computerized working memory training with game elements enhance motivation and training efficacy in children with ADHD?. Cyberpsychol Behav Soc Netw.

[12] Rapport MD, Scanlan SW, Denney CB (1999). Attention-deficit/hyperactivity disorder and scholastic achievement: a model of dual developmental pathways. J Child Psychol Psychiatry.

[13] Baddeley A (2003). Working memory: looking back and looking forward. Nat Rev Neurosci.

[14] Baddeley A (2007). Working memory, thought, and action: OUP Oxford.

[15] Hulme C, MacKenzie S, Hillsdale (NJ) (1992). Working memory and severe learning difficulties.

[16] Siegel LS, Ryan EB (1989). The development of working memory in normally achieving and subtypes of learning disabled children. Child Dev.

[17] Rogers M, Hwang H, Toplak M, Weiss M, Tannock R (2011). Inattention, working memory, and academic achievement in adolescents referred for attention deficit/hyperactivity disorder (ADHD). Child Neuropsychol.

[18] Gathercole SE, Pickering SJ, Knight C, Stegmann Z (2004). Working memory skills and educational attainment: Evidence from national curriculum assessments at 7 and 14 years of age. Applied Cognitive Psychology.

[19] Bull R, Johnston RS, Roy JA (1999). Exploring the roles of the visual‐spatial sketch pad and central executive in children's arithmetical skills: Views from cognition and developmental neuropsychology. Dev Neuropsychol.

[20] Gathercole S, Alloway TP (2008). Working memory and learning: A practical guide for teachers: Sage.

[21] Holmes J, Gathercole SE, Place M, Dunning DL, Hilton KA, Elliott JG (2010). Working memory deficits can be overcome: Impacts of training and medication on working memory in children with ADHD. Appl Cognit Psychol.

[22] Ivarsson M, Strohmayer S (2010). Working memory training improves arithmetic skills and verbal working memory capacity in children with ADHD.

[23] Klingberg T, Fernell E, Olesen PJ, Johnson M, Gustafsson P, Dahlstrom K (2005). Computerized training of working memory in children with ADHD--a randomized, controlled trial. J Am Acad Child Adolesc Psychiatry.

[24] Truedsson E, Strohmayer S (2010). Working Memory Training - theory and practice.

[25] Schweizer S, Hampshire A, Dalgleish T (2011). Extending brain-training to the affective domain: increasing cognitive and affective executive control through emotional working memory training. PLoS One.

[26] Chase WG, Ericsson KA, Faloon S (1980). Acquisition of a memory skill DTIC Document.

[27] Klingberg T, Forssberg H, Westerberg H (2002). Training of working memory in children with ADHD. Journal of clinical and experimental neuropsychology.

[28] Loomes C, Rasmussen C, Pei J, Manji S, Andrew G (2008). The effect of rehearsal training on working memory span of children with fetal alcohol spectrum disorder. Res Dev Disabil.

[29] Douglas VI, C. QH, E. HA (1999). Cognitive control processes in attention deficit hyperactivity disorder. Handbook of disruptive behavior disorders.

[30] Douglas VI, Parry PA (1983). Effects of reward on delayed reaction time task performance of hyperactive children. J Abnorm Child Psychol.

[31] Sagvolden T, Johansen EB, Aase H, Russell VA (2005). A dynamic developmental theory of ADHD predominantly hyperactive/ impulsive and combined type. Behav Brain Sci.

[32] Shanahan MA, Pennington BF, Willcutt EW (2008). Do motivational incentives reduce the inhibition deficit in ADHD?. Dev Neuropsychol.

[33] Luman M, Oosterlaan J, Sergeant JA (2005). The impact of reinforcement contingencies on AD/HD: a review and theoretical appraisal. Clin Psychol Rev.

[34] Sergeant JA, Oosterlaan J, van der Meere J (1999). Information processing and energetic factors in attention-deficit/hyperactivity disorder Handbook of disruptive behavior disorders.

[35] Barkley RA (1997). Behavioral inhibition, sustained attention, and executive functions: constructing a unifying theory of ADHD. Psychol Bull.

[36] Shaw R, Lewis V (2005). The impact of computer‐mediated and traditional academic task presentation on the performance and behaviour of children with ADHD. Journal of Research in Special Educational Needs.

[37] Bayerl M, Dielentheis TF, Vucurevic G, Gesierich T, Vogel F, Fehr C (2010). Disturbed brain activation during a working memory task in drug-naive adult patients with ADHD. Neuroreport.

[38] Kobel M, Bechtel N, Specht K, Klarhofer M, Weber P, Scheffler K (2010). Structural and functional imaging approaches in attention deficit/hyperactivity disorder: does the temporal lobe play a key role?. Psychiatry Res.

[39] Koepp MJ, Gunn RN, Lawrence AD, Cunningham VJ, Dagher A, Jones T (1998). Evidence for striatal dopamine release during a video game. Nature.

[40] Lawrence V, Houghton S, Tannock R, Douglas G, Durkin K, Whiting K (2002). ADHD outside the laboratory: Boys' executive function performance on tasks in videogame play and on a visit to the zoo. J Abnorm Child Psychol.

[41] Hinshaw SP (1992). Academic underachievement, attention deficits, and aggression: comorbidity and implications for intervention. J Consult Clin Psychol.

[42] Barkley RA (1998). Attention-deficit hyperactivity disorder: A handbook for diagnosis and treatment.

[43] American Psychiatric Association (2000). Diagnostic and statistical manual of mental disorders.

[44] Achenbach TM, Rescorla LA (2001). Manual for the ASEBA School-Age Forms and Profiles.

[45] Minaei A (2006). Confirmatory factorial analysis of Achenbach’s teacher report form. Research on exceptional children.

[46] Minaei A (2006). Adaptation and Standardization of Achenbach’s teacher report form, self-report form, teacher report form. Research on Exceptional children.

[47] Wechsler D, Kaplan E, Fein D (2004). Wechsler Intelligence Scale for Children Fourth Edition-Integrated: Technical and interpretative manual.

[48] Pham AV, Hasson RM (2014). Verbal and visuospatial working memory as predictors of children's reading ability. Arch Clin Neuropsychol.

[49] Rapport MD, Alderson RM, Kofler MJ, Sarver DE, Bolden J, Sims V (2008). Working memory deficits in boys with attention-deficit/hyperactivity disorder (ADHD): the contribution of central executive and subsystem processes. J Abnorm Child Psychol.

[50] Dehn MJ (2011). Working memory and academic learning: Assessment and intervention.

[51] Shiffrin RM, Izawa C (1999). 30 years of memory. On human memory: Evolution, progress, and reflections on the 30th anniversary of the Atkinson-Shiffrin model Mahwah.

[52] Gathercole SE (1999). Cognitive approaches to the development of short-term memory. Trends Cogn Sci.

[53] Richardson JTE, Richardson JTE, Engle RW, Hasher L, Logie RH, R. SE, Zacks RT (1996). Evolving concepts of working memory. Working memory and human cognition.

[54] Carlson RA, Khoo BH, Yaure RG, Schneider W (1990). Acquisition of a problem-solving skill: Levels of organization and use of working memory. J Exp Psychol-Gen.

[55] Davies GM (1980). Can memory be educated?. Educ Stud.

[56] Cook NM (1989). The applicability of verbal mnemonics for different populations: A review. Appl Cogn Psychol.

[57] Congos D (2005). 9 types of mnemonics for better memory.

[58] Mastropieri MA, Scruggs TE, Levin JR (1985). Maximizing What Exceptional Students Can Learn A Review of Research on the Keyword Method and Related Mnemonic Techniques. Remedial and Special Education.

[59] Castellanos FX, Tannock R (2002). Neuroscience of attention-deficit/hyperactivity disorder: the search for endophenotypes. Nat Rev Neurosci.

[60] Baddeley AD, J. PS (2006). Working memory: An overview. Working memory and education.

[61] Clair-Thompson HL (2007). The influence of strategies on relationships between working memory and cognitive skills. Memory.

[62] Hutton UM, Towse JN (2001). Short-term memory and working memory as indices of children's cognitive skills. Memory.

[63] Swanson HL, Beebe-Frankenberger M (2004). The relationship between working memory and mathematical problem solving in children at risk and not at risk for serious math difficulties. Journal of Educational Psychology.

[64] Andersson U, Lyxell B (2007). Working memory deficit in children with mathematical difficulties: A general or specific deficit?. J Exp Child Psychol.

[65] Imbo I, Vandierendonck A (2007). The development of strategy use in elementary school children: working memory and individual differences. J Exp Child Psychol.

[66] Imbo I, Vandierendonck A, Vergauwe E (2007). The role of working memory in carrying and borrowing. Psychol Res.

[67] Feifer SG, DeFina PD (2000). The neuropsychology of reading disorders: Diagnosis and intervention workbook.

